# Supplementary material for: Splice-Junction-Based Mapping of Alternative Isoforms in the Human Proteome
Source: Cell Rep. Author manuscript; Available in PMC 2020 Jan 15. (PMC6961840; doi:10.1016/j.celrep.2019.11.026)
Supplement: 3 [file NIHMS1546469-supplement-3.zip › DF2/PXD0010154/Spleen-136-Q9NYU2-KDDSFIRPVTF.pdf]

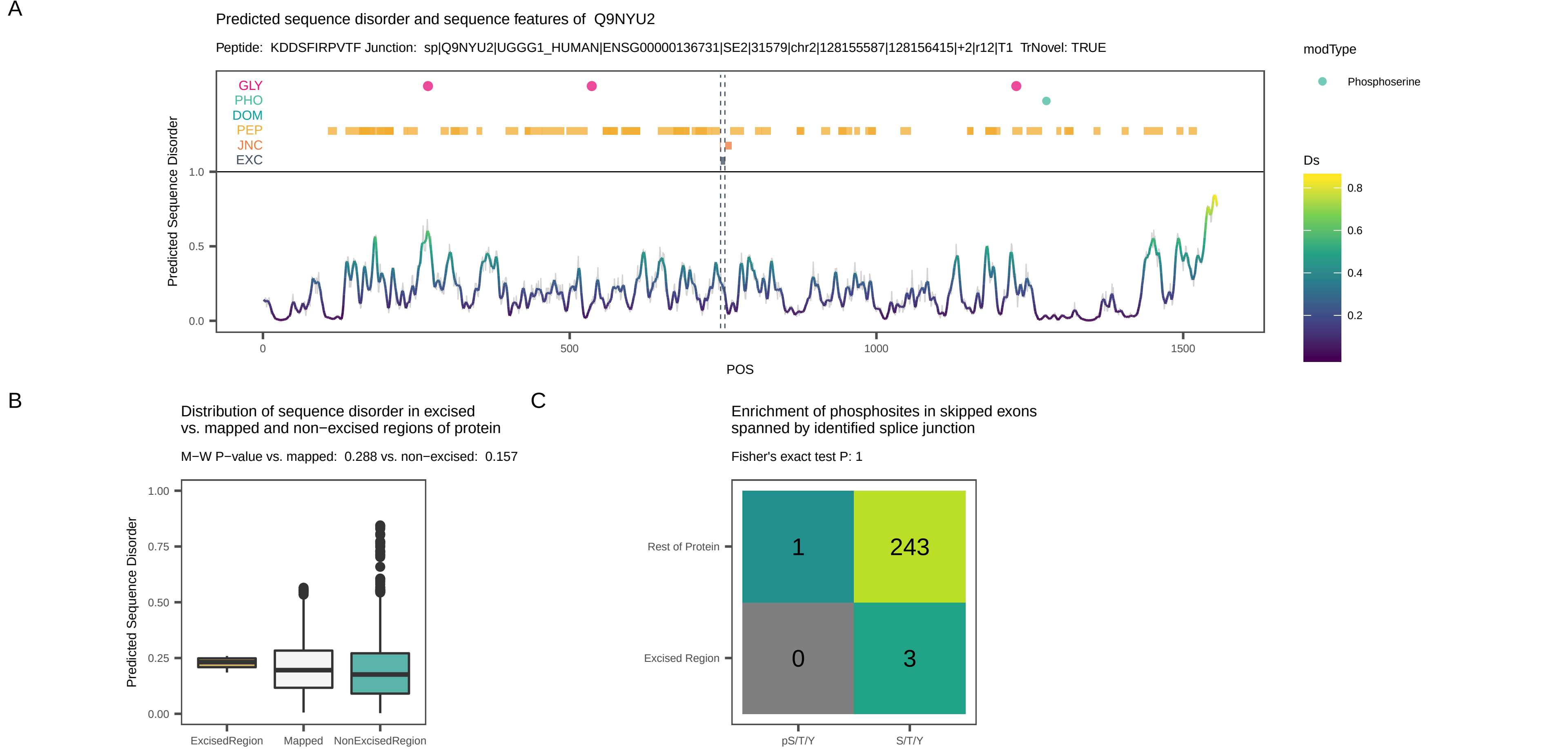

A
Predicted sequence disorder and sequence features of Q9NYU2
Peptide: KDDSFIRPVTF Junction: sp|Q9NYU2|UGGG1_HUMAN|ENSG00000136731|SE2|31579|chr2|128155587|128156415|+2|r12|T1 TrNovel: TRUE
modType
Phosphoserine
GLY
PHO
DOM
PEP
JNC
EXC
Predicted Sequence Disorder
1.0
0.5
0.0
0
500
1000
1500
POS
Ds
0.8
0.6
0.4
0.2
B
Distribution of sequence disorder in excised
vs. mapped and non−excised regions of protein
M−W P−value vs. mapped: 0.288 vs. non−excised: 0.157
Predicted Sequence Disorder
1.00
0.75
0.50
0.25
0.00
ExcisedRegion
Mapped
NonExcisedRegion
C
Enrichment of phosphosites in skipped exons
spanned by identified splice junction
Fisher's exact test P: 1
Rest of Protein
Excised Region
1
243
0
3
pS/T/Y
S/T/Y
